# Diversity in the bone marrow niche: Classic and novel strategies to uncover niche composition

**DOI:** 10.1111/bjh.18355

**Published:** 2022-07-15

**Authors:** Raúl Sánchez‐Lanzas, Foteini Kalampalika, Miguel Ganuza

**Affiliations:** ^1^ Centre for Haemato‐Oncology, Barts Cancer Institute Queen Mary University of London London UK

**Keywords:** bone marrow niche, cellular interactomes, haematopoietic stem cells, murine models, single‐cell technologies

## Abstract

Our view on the role and composition of the bone marrow (BM) has dramatically changed over time from a simple nutrient for the bone to a highly complex multicellular tissue that sustains haematopoiesis. Among these cells, multipotent haematopoietic stem cells (HSCs), which are predominantly quiescent, possess unique self‐renewal capacity and multilineage differentiation potential and replenish all blood lineages to maintain lifelong haematopoiesis. Adult HSCs reside in specialised BM niches, which support their functions. Much effort has been put into deciphering HSC niches due to their potential clinical relevance. Multiple cell types have been implicated as HSC‐niche components including sinusoidal endothelium, perivascular stromal cells, macrophages, megakaryocytes, osteoblasts and sympathetic nerves. In this review we provide a historical perspective on how technical advances, from genetic mouse models to imaging and high‐throughput sequencing techniques, are unveiling the plethora of molecular cues and cellular components that shape the niche and regulate HSC functions.

## INTRODUCTION

**FIGURE 1:**
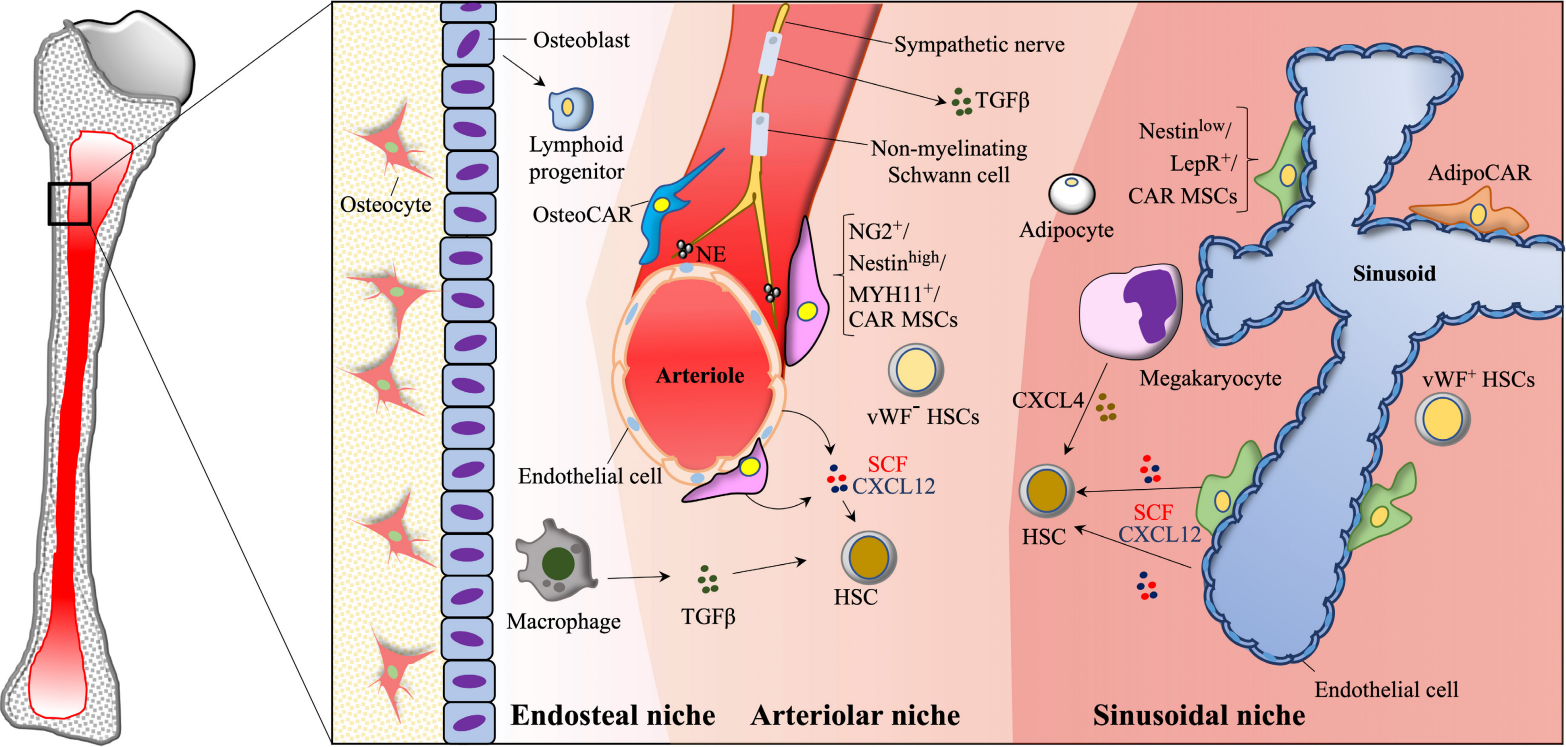
Schematic of the bone marrow (BM) microenvironment and its main regulatory components. The highly specialised BM niche supports and regulates the functions and dynamics of haematopoietic stem cells (HSCs) and other haematopoietic progenitors. Cumulative research indicates that arterioles, sinusoids and associated perivascular stromal cells play a key role in HSC maintenance. Besides endothelial cells, which produce stem cell factor (SCF) and CXC‐chemokine ligand 12 (CXCL12), vascular niche components include mesenchymal stem cells (MSCs) such as periarteriolar NG2^+^ cells, Nestin^high^ cells, MYH11^+^ cells, CXCL12‐abundant reticular (CAR) cells, perisinusoidal LepR^+^ cells and Nestin^low^ cells. Of note, there is a significant overlap between these MSCs populations. Recently described Osteo‐CAR and adipo‐CAR cells produce the highest cytokine levels in the BM and preferentially localise close to arteriolar and sinusoidal niches, respectively. Sympathetic nerves, through the release of noradrenaline (NA), are also important regulators of the HSC niche. Non‐myelinating Schwann cells produce transforming growth factor β (TGFβ) controlling HSC quiescence. The role of osteoblasts in HSC regulation is controversial but they may regulate lymphoid progenitors. Macrophages and megakaryocytes are mature haematopoietic cell types that control HSC behaviour via the release of TGFβ and CXCL4 respectively. Further studies are required to elucidate the role of adipocytes. Imaging studies of HSC subsets have demonstrated the existence of different bone marrow niches for platelet‐myeloid‐biased von Willebrand factor (*VWF* ^+^ HSCs and lymphoid biased (*VWF* ^−^) HSCs. In the figure, the endosteal and vascular niches (arteriolar and sinusoidal) are highlighted by changes in the colour of the background. For simplicity, only relevant HSC niche components are shown. Other structural components as different fibroblast‐like cells with unknown roles as HSC niche components are not included.

Haematopoietic stem cells (HSCs) sustain multilineage haematopoiesis during the lifetime of an organism. Adult HSCs reside in dedicated bone marrow (BM) niches, which sustain their functions. Extensive efforts have been devoted into unveiling the HSC niche due to its medical relevance.^1^, ^2^, ^3^, ^4^, ^5^, ^6^ The BM constitutes a highly vascularised organ. Anatomically two major BM‐niche regions can be distinguished: the vascular niche (including arterioles and sinusoids that converge in the central vein) and the endosteal niche, more closely associated with the bone^7^ (Figure 1). Within the vascular niche, besides endothelial cells (ECs), which are an intrinsic part of the blood vessels, the stromal cells provide a tri‐dimensional scaffold to the BM and include mesenchymal stem cells (MSCs), which are multipotent cells with the ability to differentiate into osteocytes, adipocytes and chondrocytes. MSCs comprise periarteriolar NG2^+^ cells, Nestin^high^ cells, MYH11^+^ cells, CXCL12‐abundant reticular (CAR) cells, perisinusoidal LepR^+^ cells and Nestin^low^ cells, amongst others.^1^, ^2^, ^3^, ^4^, ^5^, ^7^ In addition, sympathetic nerves and non‐myelinating Schwann cells associate with arteries, while megakaryocytes (MKs) connect with sinusoids. In the endosteal niche, macrophages, osteoblasts and osteoclasts do not normally associate with the vasculature.^7^ Interestingly, HSCs locate to specific areas within the BM. Particularly, HSCs concentrate close to the vasculature during homeostasis and closer to the endosteum after transplantation.^8^ Different cell types have been implicated as components of the HSC niche, such as sinusoidal endothelium, perivascular stromal cells, macrophages, MKs, osteoblasts and sympathetic nerves^1^, ^2^, ^3^, ^4^, ^5^ (Figure 1). The cellular composition of the BM dramatically changes with age,^9^ upon drug treatments (e.g. chemotherapy)^2^, ^10^ and during disease (e.g. leukaemia).^3^, ^11^, ^12^, ^13^

Anatomical and histological analyses of bone preparations provided the first insights into the function and cellular components of the BM.^14^ Modern molecular biology and genetic models enabled more sophisticated approaches to study the BM niche involving: (i) depletion and expansion of candidate niche cells, (ii) genetic deletion of essential supportive factors from candidate cell types, (iii) stem cell transplantation and (iv) high‐resolution imaging techniques^1^, ^4^, ^5^, ^7^, ^8^, ^12^ (Table 1). Furthermore, recent broader implementation of single‐cell sequencing techniques has exposed a tremendous cell diversity within the BM.^2^, ^3^, ^6^, ^15^

**TABLE 1:** Summary of the methods used to study the bone marrow niche

Strategy	Findings	Advantages	Disadvantages	References
Anatomical and histological studies	Identified a variety of cell types, suggested the existence of stem cells and the importance of the marrow in blood production.	Information on tissue architecture.	Limited functional and molecular information and difficulty to address the identity of specific cell types.	27, 28, 29, 30, 31, 32, 33
Transplantation of stromal components	Defined the presence of a haematopoietic supportive microenvironments able to recruit and support host haematopoietic activity.	Molecular and functional information.	Lack of the full plethora of steady‐state bone marrow‐niche components.	23, 24, 35, 36, 37, 38, 39, 40, 41, 43
Expansion and depletion of candidate niche cells by drug treatments	Provided insights into the role played by some niche components (e.g., osteoblasts) in the maintenance of HSCs.	Functional information.	Lack of spatial information. Broad and non‐specific side‐effects affecting multiple molecular and cellular targets that complicate the interpretation of the results.	46, 48, 49, 50, 51, 52, 53
Expansion and depletion of candidate niche cells via genetic manipulation	Explored the role of niche cells (e.g., CAR cells, megakaryocytes and macrophages) in a more specific fashion than drug treatments.	Molecular and functional information.	Lack of specificity of most promoters complicates the identification of defined candidate niche cells. The use of various promoters is advised to achieve solid conclusions. Global changes in the numbers of cells in the BM may indirectly lead to the activation of other mechanisms to regain homeostasis.	7, 21, 22, 41, 51, 54, 56, 58, 59, 60, 61, 62, 63, 64, 65, 66
Genetic deletion of essential supportive factors in the niche	Identified cellular sources of key cytokines and unveiled HSC niche components and their role regulating HSC dynamics, quiescence and self‐renewal.	Molecular and functional information.	Lack of spatial information. Promiscuous promoters target non‐specific populations complicating conclusions. The use of inducible models should be favoured over constitutive models to discern among embryonic and adult effects.	69, 70, 71, 72
2D Imaging	The use of HSC and stromal cell markers combined with genetic tracing allowed a simpler and specific identification of populations of interest.	Spatial information.	Conclusions based on limited number of cell layers. Lack of functional information. Biased by the selection of the markers used to define cell types.	8, 9, 41, 47, 69, 77, 79, 81, 82, 84
3D Imaging	Statistical analysis on the distribution of candidate niche cells and HSCs. Provided a comprehensive quantitative picture on the abundance of niche components.	Quantitative spatial and molecular information.	Lack of functional information and limited by the number of antibodies that can be employed at one single time.	61, 83, 85, 86, 87, 88
Intravital microscopy	Single‐cell resolution imaging in live animals.	Spatial, cell behavioural and longitudinal information.	Reduced molecular information and limited by the number of used markers (e.g., via genetic fluorescent labelling).	8, 13, 41, 47, 69, 70, 83, 85, 89, 90, 91, 92, 93, 94, 95, 96, 97
Single‐cell sequencing techniques	Uncovered the diversity and heterogeneity of the BM and allowed to characterise the identity of different BM cells.	Molecular and functional insights from specific and defined subsets of marrow populations.	Lack of spatial information. Insufficient sequencing depth to capture rare populations in bulk samples.	2, 3, 6, 15, 103
Spatially resolved transcriptomics	Locate transcriptionally profiled cells to particular areas previously dissected via laser‐capture microdissection.	Molecular and spatial information from specific and defined subsets of marrow populations.	Lack of information on proteomic profiles and cannot establish cell–cell interactions but only predicts them.	6, 105, 106, 107
Fluorescent labelling of cells in spatial proximity	Allowed labelling of neighbouring cells and the study of the cellular environment (e.g., in leukaemia, AML).	Spatial information of the cells of interest and prospective isolation and characterisation of niche cells.	Lack of functional information. Unable to distinguish whether cells are in cell–cell contact or distant.	108, 109

Abbreviations: AML, acute myeloid leukaemia; BM, bone marrow; CAR, CXCL12‐abundant reticular (cells); (2)(3)D, (two) (three)‐dimensional; HSC, haematopoietic stem cell.

These approaches are limited by one or more of these factors^7^: (i) infidelity of Cre‐recombinase (CRE) expression (e.g., lack of specificity for subsets of mesenchymal cells and contradictory results on the role of osteoblasts^16^), (ii) low‐resolution, (iii) low‐throughput, (iv) inability to label and visualise niche cells of unknown phenotype, and (v) insufficient sequencing depth to capture the small number of cells that are in cell–cell contact with the rare HSC population.

Thus, although a remarkable body of data has been produced, the exact composition of the niches that harbour and maintain HSCs and haematopoietic stem and progenitor cells (HSPCs) are still a matter of intense debate. In this review, we focus on the niches that support HSCs as the most extensively studied and on mouse as the most widespread mammalian model of haematopoiesis (Table 2). We provide a historical perspective on how our understanding of the structure and cellular composition of the BM niche has evolved over time based on the implementation of new approaches. We describe in detail these research strategies, their technical limitations and advantages, what was learnt from them and how new methods complement previous techniques to elucidate the cellular interactomes in the BM^1^, ^2^, ^3^, ^4^, ^5^, ^6^, ^15^ (Table 1).

**TABLE 2:** Mouse strains used to study the bone marrow niche

Strategy	Mouse strain	Description	Major findings	References
‘HSC‐reporter’ mouse strains	*Mds1‐GFP*	GFP‐labelling of haematopoietic stem and progenitor cells (HSCs and MPPs).	LT‐HSCs localise close to sinusoidal blood vessels and the endosteal surface. MPPs locate closer to transition zone vessels in intravital imaging studies in calvarium.	95
	*Flt3‐Cre; Mds1‐GFP*	GFP^+^ LT‐HSCs: CRE expression in differentiating HSCs ablates floxed GFP in MPPs limiting GFP expression to LT‐HSCs.		
	*Pdzk1ip‐CreERT; R26LoxStopLox‐tdTom*	Tamoxifen‐inducible expression of tdTomato in HSCs.	Significant motility of tdTomato^+^ HSCs in the perivascular space. Intermittent contacts with SCF‐expressing perivascular stromal cells.	97
	*HoxB5‐tri‐mCherry*	mCherry‐labelling of long‐term HSCs.	mCherry^+^ HSCs associated with VE‐cadherin^+^ endothelial cells.	93
	*α‐Catulin‐GFP*	GFP‐labelling of HSCs.	α‐Catulin–GFP^+^ c‐Kit^+^ HSCs preferentially locate to the central marrow in close contact with LepR^+^ and CXCL12^+^ niche cells.	85, 92
	*VWF‐eGFP*	GFP‐labelling of myeloid‐biased HSCs.	Myeloid‐biased and lymphoid‐biased HSCs occupy distinct BM niche microenvironments that are differentially regulated.	94
	*SCL‐tTA; H2BGFP*	Doxycycline‐inducible labelling of HSPCs with Histone H2B‐GFP.	DOX‐pulsing visualises dormant and non‐dormant HSPCs which show similar localisation in the BM.	85
	*Rosa26‐M2‐rtTA; TetOP‐H2B‐GFP*	Doxycycline‐inducible labelling of HSCs with Histone H2B‐GFP. Most functional HSCs contained within H2B‐GFP^+^ c‐Kit^+^ cells	Abundance of empty HSC niches available for engraftment upon transplantation of non‐conditioned recipients.	98
‘Stroma‐reporter’ mouse strains	*Nestin‐GFP*	GFP‐labelling of mesenchymal stem cells.	Studies of 3D‐imaging describe two subtypes of Nestin‐GFP cells in the BM: NG2^+^ Nestin‐GFP^BRIGHT^ stromal pericyte cells in arterioles and LepR^+^ Nestin‐GFP^low^ in sinusoids.	83
	*Scf‐GFP*	GFP‐labelling of SCF expressing cells including CXCL12‐expressing perivascular stromal and endothelial cells.	Scf‐GFP^+^ cells express high levels of mesenchymal stem/stromal cell markers and localise close to HSCs.	69
	*Cxcl12‐DsRed* and *Cxcl12‐GFP*	Constitutive fluorescent labelling of CXCL12‐expressing cells.	Brightest DsRed and GFP‐expressing cells include perivascular stromal cells and endothelial cells. Distributed around sinusoids	47, 70
	*Col2*.*3‐GFP; Cxcl12‐DsRed*	GFP^+^ osteoblasts cells. DsRed^+^ CXCL12‐expressing cells.	Osteoblasts express CXCL12.	70
	*Scf‐GFP; Cxcl12‐DsRed*	GFP^+^ SCF‐expressing cells. DsRed^+^ CXCL12‐expressing cells.	In the perivascular stroma all Scf‐GFP^+^ cells are Cxcl12‐DsRed^+^ and vice versa.	70
	*Lepr–Cre; loxP– EYFP*	YFP^+^ LepR‐expressing perivascular stromal cells.	LepR^+^ perivascular stromal cells do not express Nestin but express mesenchymal stem/stromal cell markers including CXCL12, PDGFRα and PDGFRβ.	69, 70
	*mCol2*.*3‐PPR*	Exogenous PPR expression in osteoblasts.	Increased numbers of osteoblasts leading to accumulation of HSPCs via activation of NOTCH1 receptor in HSCs.	21
‘Stroma‐deleter’ mouse strains	*Nestin‐CreERT2; iDTR*	Tamoxifen/Diphtheria toxin‐ induced ablation of Nestin^+^ MSCs.	Reduction in CD150^+^ CD48^−^ LSK cells in the BM. No effect on global BM and Lin^−^ CD48^−^ cellularity.	41
	*Cxcl4‐cre; iDTR*	Diphtheria toxin‐induced ablation of megakaryocytes.	Increased HSC numbers with no effect in the number of osteolineage cells, Nestin^dim^, CD51^+^ PDGFR‐α^+^ perivascular cells and Nestin^high^ cells.	61
	*Pf4‐Cre; iDTR*	Diphtheria toxin‐induced ablation of megakaryocytes. Potential promiscuous Cre expression in HSCs.	Reduction in the number of HSCs and repopulating units in the BM. Potential unspecific effects as expression of PF4 in HSCs cannot be ruled out.	62, 63
	*Pf4‐Cre; Mos‐iCsp3*	AP20187‐induced ablation of megakaryocytes. Promiscuous Cre expression in HSCs is possible.		
	*ratCol2*.*3‐TK*	Ganciclovir‐inducible ablation of osteoblasts.	Global loss of bone marrow cellularity, including lymphoid, myeloid and erythroid progenitors and HSCs. Specificity problems due to ‘bystander killing’.	56, 57
	*Macrophage Fas‐induced apoptosis (Mafia)*	AP20187‐inducible ablation of *c‐fms* expressing cells (macrophages) through a suicide fusion protein.	Loss of osteocalcin‐positive osteoblasts and HSC mobilisation. *Mafia* transgene is also expressed in CD11b^+^ and Ly‐6G^+^ myeloid cells.	51
	*Cxcl12‐DTR‐GFP*	Diphtheria toxin‐inducible ablation of CAR cells (suicide gene).	Ablation of adipogenic and osteogenic differentiation potential of marrow cells and SCF and CXCL12 production in the BM. Reduction in HSCs number and cell size.	59
	*Cxcl4‐cre; iDTR; VWF‐eGFP*	Diphtheria toxin‐inducible ablation of megakaryocytes.	Selective increase in the number of VWF^+^ HSCs.	94
	*Dmp‐1‐DTR*	Diphtheria toxin‐inducible ablation of osteocytes.	Resistant to G‐CSF‐induced HSC mobilisation	66
‘Niche factors‐deleter’ mouse strains	*Mx1‐Cre; Cxcr4* ^*flox/null*^	pIpC‐inducible ablation of *Cxcr4* in BM MSCs, Nestin^+^ cells and perivascular cells.	Severe reduction in HSC numbers. Promiscuous expression of *Mx1–Cre* complicates interpretation of results.	16, 47
	*Vav‐Cre; Scf* ^*fl/*−^	*Scf* ablation in haematopoietic cells.	*No effect in* the numbers and function of HSCs in the BM.	69
	*mCol2*.*3‐Cre; Scf* ^*fl/*−^	*Scf* ablation in osteoblasts.		
	*Nestin‐Cre; Scf* ^*fl/*−^	*Scf* ablation in mesenchymal stem cells.		
	*Nestin‐CreERT; Scf* ^*fl/fl*^	Tamoxifen‐inducible ablation of *Scf* in mesenchymal stem cells.		
	*Lepr‐Cre; Scf* ^*fl/gfp*^	Constitutive *Scf* ablation in perivascular stromal cells.	Reduction in the number of HSCs.	69
	*Ubc‐CreERT; Scf* ^*fl/fl*^	Tamoxifen‐inducible ubiquitous *Scf* ablation.	Decreased cellularity in the bone marrow and spleen and depletion of HSCs.	69
	*Tie2‐Cre; Scf* ^*fl/*−^	*Scf* genetic ablation in endothelial cells.	Reduction in the number of HSCs and haematopoietic defects during embryonic development. This highlights the importance of using TAM‐inducible *Cre‐ERT/Cre‐ERT2* models to distinguish among embryonic and adult BM specific defects.	69
	Vav‐Cre; Cxcl12^*fl/fl*^	*Cxcl12* genetic ablation in haematopoietic cells.	No effect on the function and numbers of HSCs and HSPCs.	70, 71
	*Nestin‐Cre; Cxcl12* ^*fl/fl*^	Constitutive ablation of *Cxcl1*2 in mesenchymal stem cells.		
	*Oc‐Cre; Cxcl12* ^*fl/fl*^	*Cxcl12* ablation in mature mineralising osteoblasts.		
	*Tie2‐Cre; Cxcl12* ^*fl/fl*^	*Cxcl12* ablation in endothelial cells.	Modest reduction in the number of phenotypic and transplantable HSCs in the BM without increasing HSC numbers in the blood.	70, 71
	*Prx1‐Cre; Cxcl12* ^*fl/fl*^	*Cxcl12* ablation in mature osteoblasts, osteocytes, CAR cells and CD45^−^Lin^−^ PDGFRα^+^SCA1^+^Nestin^−^LepR^−^ mesenchymal progenitors.	Additive phenotype among *Col2*.*3‐Cre; Cxcl12* ^*fl/fl*^ and *Lepr‐Cre; Cxcl12* ^*fl/*−^mice as a consequence of *Prx1‐Cre* expression in both perivascular stromal cells and osteoblasts.	70, 71
	*Osx‐Cre; Cxcl12* ^*fl/fl*^	*Cxcl12* ablation in mature osteoblasts, osteocytes and CAR cells.	HSPC mobilisation and loss of B‐lymphoid progenitors but normal HSC function.	71
	*Lepr‐Cre; Cxcl12* ^*fl/*−^	*Cxcl12* ablation in sinusoidal perivascular niches.	Mobilisation of HSCs into the circulation and effect on HSCs location.	70, 71, 72
	*mCol2*.*3‐Cre; Cxcl12* ^*fl/fl*^	*Cxcl12* ablation in endosteal osteoblasts.	Reduction in the number of CLPs and LMPPs. No effect in the number and engraftment potential of HSCs.	70
	*NG‐2‐Cre; Cxcl12* ^*fl/*−^	*Cxcl12* ablation in perivascular niches.	NG2^+^ Nestin^+^ arteriolar and LepR^+^ Nestin^low^ sinusoidal niches maintain HSCs in the BM. Depletion of *Cxcl12* from LepR^+^ sinusoidal cells affect HSC location.	72
	*NG‐2‐Cre‐ERT; Cxcl12* ^*fl/*−^	Tamoxifen‐inducible ablation of *Cxcl12* in arteriolar perivascular niches.		
	*Myh11‐CreERT2; Cxcl12* ^*fl/*−^	Tamoxifen‐inducible ablation of *Cxcl12* in arteriolar perivascular niches.		
	*Ng2‐Cre; Scf* ^*fl/*−^	*Scf* genetic ablation in perivascular niches.		
	*Ng2‐CreERT; Scf* ^*fl/*−^	Tamoxifen‐inducible ablation of *Scf* in arteriolar perivascular niches.		
	*Lepr‐Cre; Scf* ^*fl/*−^	*Scf* ablation in sinusoidal perivascular niches.		
	*Mx1–Cre; Bmpr1a* ^*fl/fl*^	pIpC‐inducible *Bmpr1a* ablation in BM MSCs, Nestin^+^ cells, perivascular cells, and cells in other tissues.	Increased number of osteoblasts and BrdU^−^LSK cells.	22

Abbreviations: BM, bone marrow; *Bmpr1a*, bone morphogenetic protein receptor type 1a; CAR, CXCL12‐abundant reticular cells; CLP, common lymphoid progenitor; *Col2*.*3*, collagen 2.3 promoter; *Cxcl12*, CXC‐chemokine ligand 12; *Dmp‐1*, dentin matrix protein‐1; DT, diphtheria toxin; DTR, DT receptor; GFP, green fluorescent protein; *Hoxb5*, homeobox B5; HSC, haematopoietic stem cell; HSPCs, haematopoietic stem and progenitor cells; *Lepr*, leptin receptor; LMPPs, lymphoid‐primed multipotent progenitors; *Mds1*, myelodysplastic syndrome 1; *Myh11*, myosin heavy chain 11; *Ng2*, neural‐glial antigen 2; *Oc*, osteocalcin; *Ox*, osterix; PDGFR, plateled‐derived growth factor receptor; Pf4, platelet factor 4; PPR, PTH/PTH‐related protein receptor; *Prx‐1*, paired‐related homeobox protein 1; PTH, parathyroid hormone; Scf, stem cell factor; TK, thymidine kinase, *VWF*, von Willebrand factor.

## STEM CELLS AND NICHE CELLS: MORE THAN NEIGHBOURS

In 1896 Artur Pappenheim^17^ conceptualised the term *stem cell* describing a precursor cell capable of generating other mature types of blood cells. Early experiments during the atomic age demonstrated that lead‐shielding the spleens of lethally irradiated mice prevented mortality.^18^ Stem cells were proved later to be the critical protective factor. Till and McCulloch^19^ reported that mice marrow cells injected in irradiated recipients could lead to the formation of colonies of proliferating cells with self‐renewing abilities in their spleens (CFU‐S). Noticing that CFU‐S stem cells were less robust than the cells of the BM at reconstituting haematopoiesis in irradiated animals, Ray Schofield^20^ formulated the *niche hypothesis* in which a stem cell is associated with other cells that determine its behaviour and fate. The identification of heterologous cells influencing stem/progenitor cells in mammals provided experimental evidence for this hypothesis.^21^, ^22^, ^23^, ^24^

Overall, *BM stem cell niches* can be defined as highly specialised and dynamic microenvironments that support HSPCs. Moreover, these niches integrate a variety of cues to efficiently respond to a plethora of insults, including infection and bleeding, to maintain tissue homeostasis throughout life.^4^, ^5^, ^10^ This implies balancing stem cell differentiation and self‐renewing decisions to generate the billions of blood cells required daily, while simultaneously preserving the stem cell population size and avoiding leukaemia development.^20^

## UNVEILING THE BM NICHE: EVOLUTION IN RESEARCH APPROACHES

### Anatomical and histological analyses: early studies

Numerous research methods have shaped our view on the role and composition of the HSC niches. Aristotle (384–322 BC) described the marrow as some ‘sort of bone waste byproduct’,^14^ while Hippocrates (460–375 BC) and Galen (130–200) considered it the source of nutrients for the bone.^14^, ^25^ In the 18th century, Jacques‐François‐Marie Duverney and Charles Robin noticed that bone is formed before marrow during development and that not every adult bone harbours a marrow, leading them to consider the marrow as the vascular element of the bones.^14^, ^25^, ^26^ In the 19th century, Ernst Neumann, Giulio Bizzozero (disciples of Rudolph Virchow) and William Osler described nucleated red blood cells, white blood cells and giant marrow cells (i.e. MKs) in the BM of humans and rabbits,^27^, ^28^, ^29^ leading to the modern view of the BM as the site of adult haematopoiesis.

Anatomically, Xavier Bichat (1771–1802) had already described the presence of red marrow and yellow fatty marrow.^30^, ^31^ Neumann noted that in most bones the marrow changes from red marrow at birth to a yellow adult marrow and claimed that blood production is confined to the red marrow in the central bones.^32^ Osler described the medulla of patients with leukaemia as similar to the ‘matter in the core of an abscess’ and the marrow in pernicious anaemia as comparable to the red marrow of a child.^14^, ^29^ Paul Ehrlich, using acid and basic aniline coal tar dyes, identified numerous haematopoietic cell types and classified leucocytes based on the staining properties of their granules.^33^ Remarkably, Neumann also proposed the presence of ‘great lymphozyt stem cell’ capable of both self‐renewing and producing lymphocytes and erythroid cells in the BM.^34^

Thus, early anatomical and histological studies demonstrated not only the presence of different anatomical types of BM but also identified a variety of cell types. Moreover, these pioneer studies cemented the importance of the marrow niche as the source for blood production and suggested the presence of stem cells.

### Evaluation on the ability of niche cells to transfer haematopoietic niche activity in vivo

In the 1960s, transplantation of stromal tissues provided the first experimental evidence on the presence of a haematopoietic microenvironment and on the role of niche factors. Particularly, subcutaneous marrow implantation revealed that stromal fibroblast‐like precursors are able to support and reconstitute a haematopoietic microenvironment upon autologous and heterotopic transplantation.^23^, ^24^, ^35^, ^36^

Notably, co‐transplantation of murine osteoblasts, but not dendritic cells, enhances multilineage engraftment of transplanted Lineage^−^ haematopoietic progenitor cells in lethally irradiated mice.^37^ Moreover, subcutaneous transplantation of human CD146^+^ multipotent subendothelial reticular BM cells into immunocompromised mice resulted in human‐derived bone tissue, which was colonised by murine haematopoietic progenitor cells.^38^ Similarly, foetal CD105^+^Thy1.1^−^ skeletal bone progenitors transplanted in the adult mouse kidney capsule gave rise to donor‐derived chondrocytes and recruited host haematopoietic activity, including long‐term engrafting HSCs (LT‐HSCs).^39^ Similarly, subcutaneous and *in* renal capsule transplantations of matrix‐embedded human and murine PDGFRα^+^CD51^+^ mesenchymal stem cells (MSC) rendered ectopic BM niches recruiting host‐HSCs.^40^, ^41^

Interestingly, in the *Steel‐Dickie S1/S1* ^*d*^ mutant mice stem cell factor (SCF, also known as c‐Kit ligand [KitL] and steel factor [SL]) levels are severely reduced and exhibit low HSPC numbers.^42^, ^43^ Transplantation of wild‐type (*wt*) spleen stroma into spleens of non‐irradiated *S1/S1* ^*d*^ mice locally triggered host erythropoiesis and provided some of the first evidence on the role of SCF as a niche factor.^43^

### Selective expansion and depletion of candidate niche cells

Expansion and ablation of candidate niche cells via drug treatments and genetic manipulation followed by prospective evaluation on the numbers of HSC/HSPCs offered new means to functionally evaluate the role of these cells in the HSC niche.^21^, ^22^

### Drug treatments

#### Expansion of candidate niche cells by drug treatments

Parathyroid hormone (PTH) administration in *wt* mice expands the number of osteoblasts, Nestin^+^ MSCs and Lin^−^Sca1^+^c‐Kit^+^ (LSK) HSPCs in the BM and improves survival in BM transplantation.^21^, ^41^ A primary role for the osteoblasts increasing LSK numbers was suggested; however, the multicellular effects of PTH makes it difficult to disentangle the role of those perturbed cells. Unfortunately, PTH administration after umbilical cord blood transplantation in human patients has shown no effect on blood count recovery in phase II clinical trials.^44^

Conversely, *in vivo* administration of strontium, a bone anabolic agent, increases osteoblast number (although not N‐cadherin^+^ osteoblasts), bone volume, and trabecular thickness, but does not affect HSPC numbers.^45^

#### Depletion of candidate niche cells by drug treatments

Granulocyte colony‐stimulating factor (G‐CSF) administration mobilises HSPCs to the blood circulation through complex mechanisms involving various BM cell types. Importantly, G‐CSF reduces chemokine ligand 12 (CXCL12) (i.e. stromal cell‐derived factor‐1 [SDF‐1]) levels,^46^ whose receptor, CXCR4, is highly expressed in HSCs.^47^ The CXCL12–CXCR4 axis plays a critical role in HSC trafficking and niche retention, as illustrated by a severe reduction in BM‐HSC numbers following *Cxcr4* ablation in *Mx1Cre‐Cxcr4* ^*flox/null*^ mice.^47^ Genetic and pharmacological disruption of the sympathetic system inhibits G‐CSF‐induced HSC mobilisation,^48^ suggesting a regulatory role for this system on the HSC niche. Specifically, G‐CSF increases the duration of sympathetic noradrenaline signals on β2‐ and β3‐adrenergic receptors. In BM stromal cells, activation of these receptors downregulates CXCL12 expression promoting HSC release.^46^, ^49^, ^50^

Additionally, G‐CSF simultaneously depletes a population of endosteal macrophages (osteomacs, which normally support osteoblast) and induces G‐CSF receptor‐expressing BM leucocytes to suppress osteoblasts,^51^, ^52^ obscuring their specific role.

Administration of liposome‐embedded clodronate, which specifically kills phagocytic cells as the only cells capable of engulfing liposomes, phenocopies G‐CSF effects, including HSC mobilisation and loss of osteoblasts, supporting a role for macrophages in the HSC niche.^51^

Treatments with anti‐vascular endothelial (VE)‐cadherin and vascular endothelial growth factor receptor 2 (VEGFR2) blocking monoclonal antibodies downregulate angiogenic NOTCH ligand expression in ECs and impair HSC engraftment in vivo, indicating a role for sinusoidal ECs in this context.^53^ In contrast, zoledronate treatment, which severely decreases the number of endosteal osteoclasts, does not affect HSC mobilisation or numbers.^51^

Overall, drug treatments often exhibit multiple cellular and molecular targets. Thus, it is not immediately possible to discern among direct versus indirect effects and their relevance in the BM niche. Genetic models aimed to specifically target cell types of interest are helping to elucidate the roles of particular BM‐niche components.

### Genetic‐based models

#### Expansion of candidate niche cells by genetic manipulation

As with PTH treatments, expression of a constitutive active version of the PTH/PTH‐related protein receptor (PPR) in osteoblasts (*mCol2*.*3‐PPR* mice) increases the number of osteoblasts, which produce higher levels of NOTCH‐ligand JAGGED‐1 and trigger LSK expansion via NOTCH1 receptor activation, supporting a role for osteoblasts in the HSC niche.^21^

Bone morphogenetic protein receptor, type IA (BMPRIA) expression inhibits osteoblastic lineage differentiation from mesenchymal progenitors.^54^ Accordingly, blocking BMP signalling via *Bmpr1a* depletion in *Mx1–Cre* ^+*/T*^ *;Bmpr1a* ^*fl/fl*^ mice results in increased numbers of osteoblasts and LSKs.^22^ Additionally, transplantation of *wt* LSKs into *Bmpr1a*‐depleted recipient mice induces the expansion of *wt* transplanted LSKs, supporting a non‐cell autonomous HSPC effect.^22^ However, promiscuous expression of *Mx1–Cre* (e.g., in BM MSCs, Nestin^+^ cells and perivascular cells)^16^ obscures the specific cell type responsible for LSK expansion in this context.

#### Depletion of candidate niche cells by genetic manipulation (‘suicide genes’)

Other studies have investigated the role of candidate niche cells by genetically depleting osteoblasts, Nestin^+^ MSC, CAR cells and MKs from the BM through the expression and activation of ‘suicide genes’ including the herpesvirus thymidine kinase (*TK*), the diphtheria toxin (*DT*) receptor (*DTR*) or genetically modified dimerisable Caspase genes (e.g. *FK506‐Fas*, *Mos‐iCsp3)* in the cells of interest (Table 2).

TK expression confers sensitivity to the initially non‐toxic pro‐drug ganciclovir (GCV). GCV phosphorylation by TK and subsequent phosphorylation yield triphosphate‐GCV (the active metabolite), which incorporates into the DNA causing single‐strand breaks and apoptosis.^55^ GCV treatment of *Col2*.*3‐TK* transgenic mice (*TK* under a osteoblast promoter [Rat‐*Col2*.*3*]) results in conditional ablation of osteoblasts and a global loss of BM cellularity, including HSCs/HSPCs.^56^ Importantly, not only cells expressing TK but neighbouring cells can undergo cell death by so‐called ‘bystander killing’,^57^ making this approach less specific than desired.

Macrophage Fas‐induced apoptosis *(Mafia)* transgenic mice express FK506‐FAS (a suicide fusion protein) under the *c‐fms* ‘macrophage‐specific’ promoter. Administration of AP20187 ligand induces dimerisation and activation of the suicide protein triggering FAS‐mediated apoptosis in c‐fms‐expressing cells, leading to loss of osteocalcin^+^ (Oc) osteoblasts and HSC mobilisation.^51^ *c‐fms‐Mafia* is not expressed in osteoblasts, MSCs and ECs; however, CD11b^+^ and Ly‐6G^+^ myeloid cells express it. Thus, a broader role of myeloid cells in HSC regulation cannot be formally excluded in this model.^51^

DTR expression provides an analogous strategy for selective cell lineage depletion. DTR‐expressing cells are sensitive to DT while *wt* murine cells are insensitive.^58^ Different mouse lines have been genetically engineered to express DTR.

HSCs locate close to reticular CAR cells (which express high CXCL12 levels)^47^ and to assess their role, CARs were ablated in *Cxcl12‐DTR‐GFP* mice (*DTR* knocked in the *Cxcl12* locus) through DT treatment.^59^ This abolished adipogenic and osteogenic differentiation potential of marrow cells and SCF and CXCL12 production in the BM and led to a marked reduction in HSC number^59^ implicating adipo‐osteogenic CAR cells as part of the HSC niche.

In *Cre‐inducible DTR* transgenic mouse strain (*iDTR*),^60^ CRE mediated excision of a floxed transcriptional STOP cassette yields DTR expression. In *iDTR*; *Cre‐ERT2* double transgenic mice, combined administration of tamoxifen (TAM) and DT ablates CRE‐ERT2‐expressing cells. Nestin^+^ MSCs depletion in *Nes‐CreERT2/iDTR* mice halves the numbers of CD150^+^CD48^−^LSK LT‐HSCs in the BM. Global BM and Lin^−^CD48^−^ cellularity were not affected in these mice, supporting a cell‐specific effect of Nestin^+^ cells on HSCs.^41^

Ablation of MKs has led to conflicting results. MK ablation in *Cxcl4‐cre;iDTR* mice yielded a substantial increase of CD105^+^CD150^+^ HSCs and re‐populating units,^61^ with no effect in the BM cellularity suggesting a direct effect of MKs on HSC quiescence by CXCL4 secretion.^61^ Nevertheless, MK depletion in *Pf4‐Cre;iDTR* and *Pf4‐Cre;Mos‐iCsp3* mice produced a significant reduction on HSCs and re‐populating units in the BM.^62^, ^63^ In *Pf4‐Cre;Mos‐iCsp3* mice, administration of AP20187 triggers Caspase‐induced apoptosis via homodimerisation of iCSP3.^64^ Importantly, platelet factor 4 (PF4) is reportedly expressed in HSCs, which could lead to undesired apoptosis of HSCs in *Pf4‐Cre;Mos‐iCsp3* and *Pf4‐Cre;iDTR* mice.^65^ However, *Pf4‐Cre*–lineage traced BM cells failed to reconstitute irradiated mice^63^ questioning if HSCs express PF4.

Osteocyte depletion in dentin matrix protein‐1 *(DMP‐1*)*‐DTR* transgenic mice yields a strain resistant to G‐CSF‐induced HSC mobilisation.^66^ Likewise, *klotho* hypomorphic (*kl/kl*) mice, which display osteoporosis and a disrupted osteocyte network, exhibit a lack of HSC mobilisation in G‐CSF treatments, supporting a role for osteocytes in regulating HSPC egress from the BM. The role of the osteocytes in the HSC niche may work indirectly through effects on osteoblasts and macrophages.^66^

Overall, the lack of specificity of most promoters obscures the identification of defined candidate niche cells and advises the use of various promoters to infer consistent conclusions. Additionally, ablation of large numbers of cells in the BM may indirectly activate HSPCs to regain homeostasis.^7^ Even in the event of cell‐type‐specific ablation, discerning if the effect on HSC numbers arises from direct or indirect perturbations on other niche components requires a detailed characterisation.^7^

### Inducible ablation of genes encoding niche factors

Conditional ablation of genes encoding critical niche factors such as *Cxcl12* or *Scf* by CRE‐mediated recombination in candidate niche cells followed by the evaluation of HSC numbers has been employed to unveil HSC niche components and their role in HSC regulation.

The c‐Kit receptor (c‐Kit)–SCF axis plays a critical role regulating quiescence^67^ and self‐renewal in HSCs, which express high c‐Kit levels.^46^, ^68^

Conditional floxed *Scf* alleles (*Scf* ^*fl*^) allow *Scf* genetic deletion via CRE activity.^69^ *Scf* depletion from haematopoietic cells (in *Vav‐Cre; Scf* ^*fl/*−^ mice), osteoblasts (*Col2*.*3‐Cre; Scf* ^*fl/*−^ mice) or MSCs (*Nestin‐Cre; Scf* ^*fl/*−^ and *Nestin‐CRE‐ERT; Scf* ^*fl/fl*^ mice) does not affect the numbers and function of LT‐HSCs (CD150^+^CD48^−^LSK cells) in the BM.^69^ However, HSC numbers decrease following *Scf* depletion in ECs (*Tie2‐Cre;Scf* ^*fl/*−^ mice) and perivascular stromal cells (Leptin receptor, *Lepr‐Cre;Scf* ^*fl/gfp*^ mice), supporting the role of endothelial and Leptin^+^ stromal cells as HSC‐niche components.^69^ Constitutive CRE activity can lead to haematopoietic defects during embryonic development (e.g., in *Tie2‐Cre; Scf* ^*fl/*−^ embryos) precluding a proper interpretation of defects observed during adulthood.^69^ Thus, the use of inducible TAM‐regulated CRE activity (i.e., via *Cre‐ERT* or *Cre‐ERT2*) is advisable whenever possible.

As aforementioned, the CXCR4–CXCL12 axis is critical in HSC regulation. *Cxcl12* depletion from haematopoietic cells (in *Vav‐Cre; Cxcl12* ^*fl/fl*^ mice), MSCs (*Nestin‐Cre; Cxcl12* ^*fl/fl*^ mice) and mature mineralising osteoblasts (*Oc‐Cre; Cxcl12* ^*fl/fl*^ mice) does not affect HSCS/HSPCs.^70^, ^71^ Conversely, *Cxcl12* depletion in ECs (*Tie2‐Cre; Cxcl12* ^*fl/fl*^ mice) modestly reduces the numbers of transplantable HSCs in the BM without mobilisation,^70^, ^71^ while *Cxcl12* ablation in perivascular stromal cells (*Lepr‐Cre;Cxcl12* ^*fl/*−^ mice) results in the mobilisation of HSCs into the circulation. *Prx1‐Cre; Cxcl12* ^*fl/fl*^ mice show an additive phenotype among *Col2*.*3‐Cre;Cxcl12* ^*fl/fl*^ and *Lepr‐Cre;Cxcl12* ^*fl/*−^ mice,^70^, ^71^ reflecting *Prx1‐Cre* wider expression. Deletion of *Cxcl12* from osterix (Osx)–Cre‐expressing stromal cells (*Osx‐Cre;Cxcl12* ^*fl/fl*^, comprising CAR and osteoblasts) results in constitutive HSPC mobilisation and loss of B‐lymphoid progenitors, but normal HSC function.^71^ Both *Osx–Cre* and *Prx1–Cre* transgenes drive CRE expression in mature osteoblasts, osteocytes and CARs but *Prx1–Cre* also targets CD45^−^Lin^−^PDGFRα^+^SCA1^+^Nestin^−^LepR^−^ MSCs, which seem required for HSC and common lymphoid progenitors (CLP) maintenance.^71^ Overall, *Cxcl12* deletion in BM candidate niche cells suggests perivascular endothelial, LepR^+^ stromal and Nestin^−^LepR^−^ MSCs as HSC‐niche components and that CLPs occupy an endosteal osteoblastic niche.^70^, ^71^ Accordingly, *Cxcl12* deletion from endosteal osteoblasts (*Col2*.*3‐Cre; Cxcl12* ^*fl/fl*^ mice) diminishes CLP numbers and lymphoid reconstitution potential, albeit no effect on HSCs.^70^

Additionally, ablation of *Scf* or *Cxcl12* from sinusoidal (via *Lepr‐Cre* mice), arteriolar (*Ng2‐Cre‐ERT* and *Myh11‐Cre‐ERT2*) or both (*Ng2‐Cre*) perivascular niches show that NG2^+^Nestin^+^ arteriolar and LepR^+^Nestin^low^ sinusoidal niches have a role in maintaining HSCs in the BM, and that depletion of *Cxcl12* from LepR^+^ sinusoidal cells also affects HSC location.^72^

Moreover, genetic deletion of *Ebf3* and *Foxc1* transcription factors from all mesenchymal cells (via *Prx1‐Cre*) or more specifically in CAR cells (*Lepr‐Cre* mice) has revealed that these factors are essential to maintain the BM niches for HSCs.^73^, ^74^ *Ebf3* and *Foxc1* are expressed preferentially in CAR cells. Particularly, *Ebf3* and *Foxc1* (via *Runx1* expression^75^) prevent the differentiation of CAR cells into osteoblasts and adipocytes, respectively.^73^, ^74^ This supports CAR cells as specialised professional niche cells, whose specific features and identity are actively regulated.^76^

### Imaging techniques

Unveiling the cellular structure of the BM niche via imaging studies relies on the use of highly‐specific HSC markers and of markers specific to the candidate niche cells so that co‐location can be effectively assessed.

#### Haematopoietic stem cell markers

Initial studies showed that transplanted carboxyfluorescein succinimidyl ester (CFSE)‐labelled Lineage^−^ BM cells (a broad population of HSPCs) locate closer to the endosteum, while CFSE^+^Lineage^+^ cells preferentially distribute around the central marrow.^77^ Staining for LSK phenotype labels a mixed population of HSCs and HSPCs.^78^ In vivo bromodeoxyuridine (BrdU) pulsing identifies more quiescent HSCs (i.e., CD45^+^LSK BrdU^−^),^22^ which locate around N‐cadherin^+^CD45^−^osteoblastic cells in the BM endosteum.^22^

The remarkable discovery of signalling lymphocyte attractant molecule (SLAM) markers with the ability to identify bona fide transplantable murine HSCs as about one in three CD150^+^CD48^−^LSK cells^79^, ^80^ enabled a simple antibody combination to precisely distinguish HSCs.^79^ Two‐dimensional (2D) microscopy, and other imaging approaches discussed below, support the presence of vascular niches where most Lin^−^CD48^−^CD41^−^CD150^+^ LT‐HSCs locate to extraluminal perisinusoidal spaces and associate with sinusoidal endothelium and mesenchymal CAR cells^8^, ^41^, ^47^, ^69^, ^79^, ^81^, ^82^ and a minor portion localise around arteria and arterioles vessels.^83^, ^84^

#### Three‐dimensional (3D) whole‐mount imaging

Initial studies analysed HSC localisation taking into account single BM populations and lacked resolution at tissue level. 3D whole‐mount imaging of optically cleared BM preparations coupled with simulations of randomly assigned positions has allowed for testing of the significance of the distribution of candidate niche cells to HSCs and compare them with a null distribution.^61^, ^85^, ^86^, ^87^ 3D microscopy showed that Lineage^−^CD41^−^CD48^−^c‐Kit^+^Sca1^+^ HSPCs preferentially localise in the endosteum interacting with sinusoidal and non‐sinusoidal BM microvessels.^88^ Importantly, imaged‐based quantitative spatial analysis of BM tissues revealed that sinusoidal ECs (SECs) and CAR cells are ~30‐fold more abundant than previously assumed by flow cytometry analyses.^86^ This suggests that enzymatic and mechanical methods employed for tissue dissociation prior to flow cytometry analyses are not efficient in extracting every cell type, which can lead to confounding conclusions.^86^ Moreover, high abundance of SEC and CAR cells makes them widely available in the BM for cell interactions.^86^ Other 3D‐microscopy studies have shown that CD41^+^ MKs are not randomly distributed to Lin^−^CD48^−^CD41^−^CD150^+^ HSCs in the BM sinusoids^61^ and that quiescent HSCs associate with small endosteal arterioles ensheathed by NG2^+^ pericytes.^83^ As the criteria employed to define a random distribution of dots and the methods used to test the statistical significance of cell location largely diverge among studies, this can be a source of variability and can lead to conflicting conclusions on cell interactions.^86^

#### Intravital microscopy (IVM)

Intravital microscopy combines high‐resolution confocal microscopy and two‐photon video imaging. It allows longitudinal in vivo studies of cellular dynamics including cell migration, division, death and cell–cell interactions.^13^ IVM has exposed that LT‐HSCs preferentially locate in the endosteum close to osteoblasts and near perivascular Nestin‐GFP^+^ following transplantation into immunoablated and immunocompromised mice.^8^, ^41^, ^89^, ^90^, ^91^ IVM has been particularly useful for longitudinal studies in leukaemia progression.^13^ Recent studies on HSC motility in the BM combining IVM and the use of HSC genetic reporters are detailed below (see ‘Haematopoietic stem cell genetic reporters’).

#### Genetic reporters for niche components

Fluorescent protein expression in niche cells has greatly facilitated their identification. Green fluorescent protein (GFP) labelling of Nestin^+^ MSC cells in *Nestin‐GFP* knock‐in mice showed that CD150^+^CD48^−^Lin^−^ HSCs localise around Nestin‐GFP^+^‐MSCs and tyrosine hydroxylase^+^ catecholaminergic fibres.^41^ Nestin‐GFP^+^ cells encompass NG2^+^ Nes‐GFP^BRIGHT^ stromal pericyte cells, which locate around BM arterioles, and LepR^+^ Nes‐GFP^DIM^, distributed around sinusoids.^83^ Both sinusoidal and arteriolar niches regulate HSC numbers with arteriolar niches controlling HSC location/retention.^72^

Delineating the c‐Kit–SCF regulatory axis, SCF‐producer GFP^+^ cells in *Scf* ^*gfp/*+^ knock‐in mice exhibit high transcript levels of MSC markers and contain *Cxcl12*‐expressing perivascular stromal cells and ECs as major SCF producers. Notably, CD150^+^CD48^−^Lin^−^ HSCs frequently localise adjacent to sinusoidal blood vessels close to Scf‐GFP^+^ cells.^69^

Interestingly, CXCL12‐DsRed^+^ and CXCL12‐GFP^+^ cells (in *Cxcl12* ^*DsRed*^ and *Cxcl12* ^*GFP*^ knock‐in mice respectively) include perivascular stromal cells and ECs distributed around sinusoids.^47^, ^70^ Osteoblasts (visualised as GFP^+^ cells in *Col2*.*3* ^*GFP*^ mice) also express CXCL12 as shown in *Col2*.*3* ^*gfp/*+^ *;Cxcl12* ^*DsRed/*+^ mice, albeit at lower levels.^70^ Remarkably, in *Scf* ^*gfp/*+^ *;Cxcl12* ^*DsRed/*+^ mice all Scf‐GFP^+^ cells are Cxcl12‐DsRed^+^ and vice versa, suggesting that perivascular cells are a major source of both factors. LepR^+^ perivascular cells are exceptionally high CXCL12 producers (EYFP^+^ cells from *Lepr–Cre;loxSTOPlox–EYFP* mice express ~15 000‐fold the mRNA levels in the whole BM).^70^

#### Haematopoietic stem cell genetic reporters

In *α‐catulin‐GFP* knock‐in mice only 0.02% of BM cells are α‐catulin–GFP^+^, all HSCs are α‐catulin–GFP^+^ and 30% of α‐catulin–GFP^+^c‐Kit^+^ cells are LT‐HSCs. Thus α‐catulin–GFP^+^ very specifically identifies LT‐HSCs. Dividing and non‐dividing α‐catulin–GFP^+^c‐Kit^+^ cells locate around LepR^+^CXCL12^+^ niche cells in perisinusoidal BM locations.^92^

Likewise, mCherry expression from the *Hoxb5* locus in *Hoxb5‐tri‐mCherry* mice is mostly limited to LT‐HSCs. Only HOXB5^+^ cells harbour LT‐engraftment potential and the majority of mCherry^+^ cells associate with VE‐cadherin^+^ perivascular ECs.^93^

Von Willebrand factor (VWF)‐eGFP expression in *VWF‐eGFP* knock‐in mice segregates platelet/myeloid‐biased HSCs (VWF‐eGFP^+^) from lymphoid‐biased HSCs (VWF‐eGFP^−^). Intriguingly, VWF‐eGFP^+^ HSCs associate with MK niches, while VWF‐eGFP^−^ HSCs mostly occupy NG2^+^ arteriolar niches, highlighting the presence of two functionally distinct niches.^94^

In *Mds1‐GFP;Flt3‐Cre* mice, a floxed *Mds1‐GFP* allele is expressed in HSCs and *Flt3‐Cre* expression in differentiating HSCs restricts GFP to LT‐HSC. IVM shows LT‐HSCs located closer to sinusoidal blood vessels and the endosteal surface while MPPs concentrate to transition zone vessels.^95^ However, another group reported (Lin^−^CD41^−^CD48^−^cKIT^−^CD150^−^FLT3^+^) MPPs residing in HSC niches.^96^ Interestingly, *Mds1‐GFP* ^+^ *;Flt3‐Cre* LT‐HSCs exhibit limited motility during steady‐state haematopoiesis in IVM.^95^ Paradoxically, in vivo imaging of tdTomato^+^ labelled HSCs in *Pdzk1ip‐Cre;loxSTOPlox‐tdTomato* mice (*Pdzk1ip‐Cre* drives HSC‐specific‐CRE expression) showed significant motility for tdTomato^+^ HSCs in the perivascular space with occasional interactions with SCF^+^ perivascular cells.^97^ Differences in the expression pattern among genetic reporters and analysed bones (calvaria vs. long bones) could explain these discrepancies.

Recently, quantitative 3D‐microscopy studies have analysed the localisation of HSCs (labelled with different strategies) in relation to four simultaneous BM components in different bones.^85^ In this report, α‐catulin‐GFP^+^ HSCs and Mds1GFP^+^/Flt3Cre HSCs located close to sinusoidal CXCL12^+^ stromal cells and MK but not to bone, adipocyte or Schwann cells.^85^ Additionally, dormant (non‐dividing) HSCs, labelled as GFP‐retaining c‐Kit^+^ cells in doxycycline (DOX)‐chased *SCL‐tTA;H2BGFP* mice, showed similar location to α‐catulin‐GFP^+^ HSCs.^85^ Importantly, HSC locations reflected the abundance of the analysed BM niche cell types rather than the presence of specific microenvironments within the analysed populations.^85^ Notably, these niche cell types are much more frequent in the BM than previously assumed.^86^ Remarkably, the transplantation of very large numbers of HSCs into non‐myeloablated recipients strikingly demonstrated that donor HSCs are able to engraft and occupy niches distant from host HSCs without replacing host HSCs as visualised by DOX‐chased *Rosa26‐M2‐rtTA; TetOP‐H2B‐GFP* labelled HSCs.^98^, ^99^ This further highlights the abundance of empty HSC niches available for engraftment.

### Single‐cell profiling of BM cells

To date, imaging techniques are still biased by preselection of antibodies and limited by how many BM niches can be simultaneously analysed. Mass cytometry, or cytometry by time of flight (CyTOF) recently unveiled 28 subsets of non‐haematopoietic cells in the BM during homeostasis.^100^ This single‐cell technique allows measurements of ~50 targets per cell and enables a detailed taxonomy of the BM niche; nevertheless, it is still restricted by the number and preselection of antibodies.

Broader implementation of single‐cell RNA‐sequencing (scRNAseq) procedures recently yielded the first transcriptional profiles of the BM at single‐cell level.^2^, ^3^, ^6^, ^15^, ^101^, ^102^, ^103^ scRNAseq provides an unbiased means with which to characterise BM cells with extraordinary precision. A total of 17 cellular subtypes were identified among non‐haematopoietic unfractionated cells (7AAD^−^Calcein AM^+^Ter119^−^CD71^−^Lin^−^), comprising MSCs, osteolineages, chondrocytes, fibroblasts, ECs and pericytes.^3^ Similarly, scRNAseq of VE‐Cadh^+^ endothelial, LepR^+^ cells and COL2.3^+^ osteoblasts fractionated from Tom^+^CD45^low^Ter119^low^ stromal cells (respectively from *VECadh‐Cre;LoxP‐tdTomato*, *Lepr‐Cre;LoxP‐tdTomato* and *Col2*.*3‐Cre;LoxP‐tdTomato* mice) identified two endothelial, four perivascular and three osteo‐lineage clusters.^2^ Within those subtypes some exhibited an HSC‐regulatory gene profile (based on *Scf* and *Cxcl12* expression) including LepR^+^MSCs derived osteolineage cells, fibroblasts and periendosteal ECs.^3^ The detection of promiscuous expression of *Lepr* mRNA in multiple cell types, warns on the interpretation of data related to *Lepr‐Cre* strains.^3^ Similarly, scRNAseq data question the exact identity of classically defined Nestin^+^ mesenchymal population.^2^, ^3^, ^6^ Importantly, there are discrepancies in the expression pattern of the endogenous *Nestin* locus and marker genes. Particularly, the patterns of expression of CRE and GFP differ between *Nestin‐GFP* and *Nestin‐Cre‐ERT2* transgenic mice^83^ and endogenous *Nestin* is not expressed in adult CAR/LepR^+^ cells.^104^ scRNAseq of tdTomato^+^ mesenchymal lineage cells isolated from endosteal BM of *Col2:tdTomato* mice revealed a novel adipogenic Perilipin^+^ population with key roles regulating marrow vasculature and bone formation.^103^

Progressive depletion of abundant cell types in the BM (i.e., major immune populations and erythroid progenitors) followed by scRNAseq served to capture rare niche cellular components and exposed 32 cell clusters.^6^ They encompass Schwann cells, smooth muscle cells, myofibroblasts, EC clusters (*Sca1* ^+^ arterial and *Emcn* ^+^ sinusoidal ECs) and nine *Pdgfra* ^+^ mesenchymal populations (chondrocytes, osteoblasts, fibroblast‐like populations, *Ng2* ^+^ *Nestin* ^+^ MSCs, and two CAR clusters).^6^ These two CAR populations, namely Adipo‐CAR (similar to *Lepr*‐Cre^+^ cells) and Osteo‐CAR cells, showed the highest cytokine levels among all BM cells.^6^

The scRNAseq data lack spatial distribution information. Circumventing this, laser‐capture microdissection coupled with sequencing (LCM‐seq or spatial transcriptomics) of BM fixed sections allows assignment of cells to particular spatial locations. Particularly, Adipo‐CARs preferentially locate to perisinusoidal endothelial areas, while Osteo‐CARs to non‐vascular regions and arteriolar endothelium.^6^, ^105^ An emerging challenge from scRNAseq databases is how to readily compare cell clusters identified by different laboratories.^102^ Even more important is to establish the functional relevance of any of these novel cellular populations, which will need to rely on genetic‐based approaches (e.g., genetic ablation of candidate cells).

To predict the likelihood of interaction among cells, various algorithms and databases (e.g., RNA magnet, CellPhone DB, NicheNet) have emerged based on the expression patterns of cell‐surface receptors and their known surface‐expressed ligands.^15^, ^105^, ^106^, ^107^ Thus, scRNAseq data can be interrogated to expose cell–cell interactions and potential niche components.

### Strategies to fluorescently label cells in cell proximity

Although progressive deletion of abundant populations enriches samples for less frequent cell populations,^6^ rare populations may be missed. Particularly, rare HSCs (~20 000 total HSCs in an adult mouse^79^) may interact with a very reduced number of bona fide niche cells, especially if HSCs show low motility.^95^ Additionally, current spatial transcriptomics lack the ability to directly capture cell–cell interactions. Tackling this, a soluble lipid‐permeable mCherry (sLP‐mCherry) protein secreted by transduced cells and which can be absorbed by neighbouring cells, allows spatial location of the producer cells and prospective isolation and characterisation of niche cells (mCherry^+^) within the bulk tissue.^108^ This strategy was recently used to analyse the early niche in contact with LP‐mCherry‐expressing human acute myeloid leukaemia (AML) leukaemic cells xenografted in immunocompromised mice by the isolation and transcriptional profiling of mCherry^+^ cells.^109^ In this regard, Table 3 provides a summary of various changes in cellular composition in the BM niche in different malignant and non‐malignant diseases and conditions.

**TABLE 3:** The bone marrow niche in malignant and non‐malignant diseases

Disease/condition	Experimental findings (BM niche)	Highlights	Experimental model‐species	References
Ageing	Telomere shortening in stromal cells limits HSC function and engraftment. MSCs lose osteogenic potential and switch into an adipogenic state. Increase in Adipo‐CAR numbers. Increased myelopoiesis, anaemia, thrombopoiesis. Loss of EMCN^+^CD31^high^ vessels and β3‐adrenergic innervation. Reduced number of sinusoidal ECs and increased vessel diameter.	Age‐related BM niche defects impact HSC functions.	Human and mouse.	117, 118
Chronic viral infections	Persistent viral infections induce cytotoxic damage, interfere with normal signalling pathways and cellular trafficking in the BM. Viral DNA is often found in the human BM (e.g., herpesviruses, hepatitis B virus, Merkel cell polyomavirus and human papillomavirus). In mice chronic infection with lymphocytic choriomeningitis virus causes long‐lasting destruction of the CAR cell network due to accumulation of activated CD8^+^ T‐cells in the BM via interferon‐dependent mechanisms decreasing HSC functionality.	Chronic viral infections perturb the BM niche leading to a decreased competitive fitness in HSCs.	Human and mouse.	119, 120, 121, 122
Aplastic anaemia (AA)	Hypoplastic, fatty BM, severe reduction in HSPCs and HSCs. Acquired AA triggered by autoimmune dysregulated CD8^+^ T‐cells. Overproduction of pro‐inflammatory cytokines including interferon γ and TNFα. In AA patients: increased numbers of TNFα producing macrophages, fewer endosteal cells, vascular and perivascular cells. MSCs from AA patients display decreased clonogenic potential and proliferation and are biased to differentiate into adipogenic lineages. Allogeneic transplantation from unmanipulated BM is preferred over PB‐derived transplantation as BM niche elements recover more efficiently, suggesting MSCs in the BM contribute to a better engraftment.	It is unclear if the alterations in the BM in AA patients contribute to AA or if they are a consequence of AA, especially in acquired AA.	Human and mouse.	123, 124, 125, 126
β‐thalassaemia	Altered bone metabolism. Osteoporosis. Osteopenia. Expansion and premature apoptosis of immature erythroid precursors in the BM. Reduced quiescence of HSCs. Compromised HSC activity. Reduced PTH levels. Transplantation of thalassaemic HSCs into *wt* mice rescues HSC activity.	HSC self‐renewal deficiency in β‐thalassaemia may be caused by an altered BM niche.	Human and mouse.	127, 128, 129
Myelodysplastic syndrome (MDS)	Human MDS cells reprogram MSCs into a transplantable niche disease. Altered inflammatory signalling in niche cells could facilitate somatic mutations, clonal selection and expansion. MDS cells preclude osteolineage differentiation of MSCs thru extracellular vesicles (EVs) resulting in defective haematopoiesis.	BM failure in MDS patients results at least partially from the differentiation block of MSCs.	Human and mouse.	130, 131, 132
Acute myeloid leukaemia (AML)	Drastic remodelling of endosteal vasculature in AML. Endosteal AML cells produce pro‐inflammatory and anti‐angiogenic factors. Loss of osteoblasts, HSCs and HSC niches. Sympathetic neuropathy blocks the differentiation of Nestin^+^ MSCs into NG2^+^ cells. Increased vascular permeability allowing HSC egress from the BM. Overall loss of BM stroma in AML mouse models. Reduced numbers and activity of osteoblasts in AML patients.	AML cells severely modify and highjack the BM niche to thrive	Human and mouse.	11, 12, 13, 133, 134, 135
Multiple myeloma (MM)	MM cells impair osteoblast differentiation via secretion of Dickkopf‐1 and IL3; activate osteoclasts by VEGF secretion; and induce angiogenesis by secreting VEGF, HGF and other cytokines. MM cells inhibit T‐cells by TGFβ and IL10 creating an immunosuppressive environment. The modified BM niche supports MM growth, disease progression and chemoresistance via a combination of secreted cytokines, chemokines and an altered extracellular matrix.	MM cells alter the cytokine milieu in the BM. The BM niche facilitates survival signals and disease progression.	Human and mouse	136, 137, 138
Chronic myeloid leukaemia (CML)	Drastic changes in the BM sinusoidal vasculature structure, increased microvascular density in CML patients. CXCL12 inhibits LSC expansion and maintains quiescence of TKI‐resistant LSCs in CML. CXCL12 depletion from MSCs enhances TKI efficacy. CXCL12‐expressing MSCs are key for preserving TKI‐resistant LSCs.	CML impacts sinusoidal vasculature.	Human and mouse.	139, 140, 141
B‐cell acute lymphoblastic leukaemia (B‐ALL)	B‐ALL blasts activate osteoclasts leading to bone resorption. Increased microvessel density via VEGF production. MSCs can trigger chemoresistance in B‐ALL thru VCAM‐1 and NOTCH‐related pathways. Increased Activin A levels inhibit CXCL12 production in MSCs. B‐ALL cells are still able to migrate towards very low CXCL12 levels, while CD34^+^ human HSCs are not. B‐ALL blasts induce MSCs to produce TGFβ promoting suppressive dendritic cells. In adult ALL, the adipocyte niche evolves during disease progression and following therapy and triggers a fate switch into quiescence in ALL cells promoting chemoresistance.	Bi‐directional influence among B‐ALL and BM niche supports B‐ALL cells	Human and mouse	142, 143

Note: This table does not provide an exhaustive list on changes observed in the BM niche under each of the indicated diseases/conditions, but rather aims to illustrate the importance of the BM niche in disease by providing relevant findings in various conditions. Effects of ageing in the BM result from multiple factors. The changes observed during ageing contribute to the development of diseases more frequently observed in the elderly as MDS and AML. Thus, some changes in the BM are expected to be shared among conditions.

Abbreviations: BM, bone marrow; CAR, CXCL12‐abundant reticular cells; EC, endothelial cell; HGF, hepatocyte growth factor; HSC, haematopoietic stem cell; HSPCs, haematopoietic stem and progenitor cells; IL, interleukin; LSC, leukaemic stem cell; MSC, mesenchymal stem cell; NG2, neural‐glial antigen 2; PB, peripheral blood; PTH, parathyroid hormone; TGFβ, transforming growth factor β; TNFα, tumour necrosis factor α; TKI, tyrosine kinase inhibitor; VCAM‐1, vascular cell adhesion molecule 1; VEGF, vascular endothelial growth factor; *wt*, wild‐type.

Of note, sLP‐mCherry producing cells label cells in proximity but cannot distinguish between distant and direct physical interactions, and transient and stable contacts.^108^, ^109^ Unveiling the type of interactions among HSCs and niche components is likely vital to define bona fide cellular and molecular cues that regulate HSCs.

## CONCLUSIONS AND FUTURE PERSPECTIVE

The development and implementation of new research techniques has dramatically changed our view of the BM from a source of nutrients for the bones to a highly specialised and complex tissue responsible for maintaining haematopoietic homeostasis.

Initial studies suggested osteoblasts as a major HSC‐niche component.^21^, ^22^, ^37^ However, more recent studies based on: (I) genetic ablation of critical molecular niche factors (mostly *Scf* and *Cxcl12*) in candidate niche cells, (II) the use of more stringent HSC markers (i.e., SLAM markers, genetic HSC reporters) and (III) sophisticated imaging techniques^8^, ^47^, ^49^, ^79^; support two major HSC niches in the BM: (i) sinusoidal niches containing ECs, MKs and CAR cells and (ii) arteriolar niches encompassing ECs, NG2^+^ pericytes, CAR cells, sympathetic nerves and non‐myelinating Schwann cells.^1^, ^4^, ^6^ Surprisingly, simultaneous imaging of HSCs and multiple BM components indicate that HSCs randomly localise within sinusoids, CXCL12^+^ stroma, and MKs.^85^ Furthermore, 3D‐quantitative microscopy indicates that these HSC niches are ~30‐times more frequent than previously assumed.^86^ Future research will determine the level of heterogeneity within the HSC niches and if some particular sub‐compartments constitute specialised subniches.

In this regard, scRNAseq technologies are exposing an unappreciated cellular diversity in the BM; nevertheless, the functional relevance of most of these populations is still to be investigated. Next steps characterising the BM niche should provide a ‘proteomic’ perspective. Recently, proteogenomic techniques based on cellular indexing of transcriptomes and epitomes by sequencing (CITE‐seq) coupled with scRNAseq have allowed mRNA and protein expression analyses at the single‐cell level.^110^, ^111^ Additionally, multiplexed imaging techniques (e.g. CODEX^112^ and IBEX CITEX^113^) render multi‐parameter high‐resolution images in tissue sections and can help to phenotypically dissect the cellular complexity in the niche.

Unveiling the type of interactions among HSCs and niche components is critical to determine the cellular and molecular signals that regulate HSCs. Novel algorithms predicting the likelihood of cellular interactions are based on known ligand receptors but ‘ignorant’ for any unknown molecular interactors. Soluble lipid‐permeable fluorescent proteins allow for identification of cells in cell proximity and enrichment for scarce cells that may not be captured by current scRNAseq techniques.^95^, ^97^, ^108^, ^109^ However, they do not provide information on the type of cellular interactions.

At this stage, only the implementation of novel unbiased methods (ideally modular and genetic) with the ability to identify and differentiate among frequent/stable cell–cell interactions versus transient and distant interactions in vivo will directly untangle the BM‐niche components. These technologies will be extremely useful in revealing the microenvironments that support any other cell of interest in any tissue (including tumour chemotherapy‐resistant cells). Neurobiology, with a long‐standing interest in uncovering synaptic partners, has employed rabies viruses, optogenetics and split forms of GFP and CFP to investigate synaptic interactions.^114^, ^115^, ^116^ The tropism of rabies viruses and nature of neurotransmitters make the first two approaches initially unsuitable to other tissues. However, split forms of GFP and CFP seem more amenable for a universal approach to detect cell–cell interactions.^115^ In summary, four decades of intense technical development and biological studies have allowed remarkable advances in our understanding of the BM niche. The foreseeable implementation of novel approaches will identify critical factors required for HSC maintenance, self‐renewal and differentiation.

## AUTHOR CONTRIBUTIONS

Raúl Sánchez‐Lanzas, Foteini Kalampalika and Miguel Ganuza wrote the manuscript.

## CONFLICT OF INTEREST

Authors declare no conflicts of interest.

## Data Availability

All the data reported here was gathered from published literature.
